# Supplementary material for: A set of multi-entry identification keys to African frugivorous flies (Diptera, Tephritidae)
Source: Zookeys. 2014 Jul 24;(428):97–108. doi: 10.3897/zookeys.428.7366 (PMC4143993; doi:10.3897/zookeys.428.7366)
Supplement: Supplementary material 6 — Key to Ceratitis [file zookeys-428-097-s006.zip › SF6_ZooKeys_key to Ceratitis/key/SF6_ZooKeys_key to Ceratitis/Media/Html/Ceratitis fulicoides .htm]

Ceratitis (Pterandrus) fulicoides (Munro)


***Ceratitis*** **(*Pterandrus*) *fulicoides*
(Munro)**

*Pterandrus fulicoides* Munro, 1943: 137.

Body
length: 5.84 (5.60-6.25) mm; wing length: 6.51 (5.80-7.00) mm.

Male

Head:
Antenna yellowish orange. First flagellomere 2-3 times
as long as pedicel. Arista with short to moderately long rays; ventral rays
shorter and sparser than dorsal rays, especially basally. Frons convex, with
poorly developed median protuberance; pale, center yellow; with short scattered
setulae largely of same color as frons. Frontal setae absent; posterior orbital
less developed. Face yellowish white. Genal seta pale or reddish; genal setulae
dark reddish, poorly developed.

Thorax:
Postpronotal lobe yellowish white, without spot. Mesonotum: ground color pale
gray with distinct orange tinge, microtrichose silvery shine; with streaks and
darker markings but without distinct spots, except at transverse suture and
around dorsocentral and prescutellar setae, prescutellar yellow-white markings
separate. Scapular setae dark. Scutellum yellowish white, basally without
distinct spots, apically with three separate black spots, extending to basal
half. Anepisternum on ventral half yellowish orange; setulae pale.

Legs:
Yellow except where otherwise noted; setation typical for subgenus, mainly
pale. Foreleg: femur posteriorly with bush of long orange setulae along entire
length, posterodorsal setulae longer; ventrally with row of long orange setae.
Midleg: femur ventrally with row of long orange setulae, on distal 0.33 closely
appressed to form feathering; midtibia with orange feathering dorsally along
distal 0.66 and ventrally along distal 0.8; anteriorly with short to moderately
long dispersed orange setulae, longer along dorsal row. Hindleg: femur at
apical 0.25 with longer setulae dorsally and to a lesser extent ventrally.

Wing:
bands brownish or yellowish brown. Marginal band only well developed on distal
half, interruption between marginal and discal bands broad and complete; discal
band narrow and partly interrupted near crossvein R-M; cubital band free; medial
band absent; crossvein R-M slightly beyond middle of discal cell. Apex of vein
R_1_ distal to level of crossvein R-M. Crossvein DM-Cu oblique
posterobasally.

Abdomen:
Mostly yellow. Tergite 2 largely and tergite 4 across posterior half with pale
gray (not silvery) band; tergite 3 across posterior 0.33 to 0.25 with brown
band, interrupted medially or with median notch; tergite 5 across posterior
margin sometimes narrowly brownish. Male epandrium in lateral view with lateral
surstylus slender and elongate; posterior lobe straight and elongate, anterior
lobe well pronounced.

Female

As
male except for the following characters: Two (sometimes three) well developed
frontal setae present; orbital setae stronger developed. Genal seta dark, genal
setulae all dark and well developed. Wing bands slightly paler; discal and
cubital bands broader and well developed; medial band weakly developed but
present, joining marginal band. Legs without feathering. Forefemur with
posterior and posterodorsal rows of dark orange setae; posteroventral setulae
pale. Ventral setae orange, basally black. Abdominal tergite 5 not darkened
posteriorly. Oviscape almost as long as preabdomen. Aculeus about eight times
longer than wide; tip pointed and lateral margin slightly concave.

(Description
after De Meyer & Freidberg, 2006)
